# Does internet use affect levels of depression among older adults in China? A propensity score matching approach

**DOI:** 10.1186/s12889-019-7832-8

**Published:** 2019-11-07

**Authors:** Yean Wang, Huan Zhang, Tong Feng, Hongyang Wang

**Affiliations:** 1 School of Social Development and Public Policy, Beijing Normal University, Room 2011 New Main Building, No.19 Xinjiekou Wai Street, Beijing, 100875 China; 2 School of Social Development and Public Policy, Beijing Normal University, Room 2014 New Main Building, No.19 Xinjiekou Wai Street, Beijing, 100875 China; 3 Beijing, China

**Keywords:** Older adults, Depression, Internet use, Propensity score matching

## Abstract

**Background:**

Emerging research on the use of new technology suggests that internet use is generally associated with high levels of efficiency among older adults in the following areas: quality of life, mood, positive psychological well-being, and the individual and societal costs of caring for them. However, there is little empirical evidence specifically concerning the causal effects of older adults’ internet use on their depression level. There is a need for more replication studies to help confirm that the emerging evidence on the impact of internet use is accurate and applicable to different populations and in different situations.

**Method:**

Using national data from the China Family Panel Study in 2016, this study helps to fill the above mentioned research gap. This study followed a two-step analytical strategy to empirically examine the association between internet use and reported depression in older adults. In the first step, we estimated a binary logistic regression model with internet use as the dependent variable and 8 demographic and socioeconomic factors as the confounding variables. In the second step, we performed a propensity score analysis to control for potential bias using the confounding variables confirmed in the first step.

**Results:**

The results show that older adults who reported internet use have lower depression levels than did those who did not use the internet, with adjustments made for gender, age, urban or rural residence, pension status, educational background, physical health, life satisfaction, and intelligence level.

**Conclusion:**

These findings suggest that it is critical to advocate for technology-based policies and programs that promote older adults’ internet use to improve their social well-being, which can also serve as a policy strategy to help alleviate older adults’ depression.

## Background

With the growth of the information technology revolution, new technologies such as the internet have penetrated all aspects of people’s lives, profoundly changing their way of life and social interactions. Older adults are also gradually becoming involved. According to American research, the proportion of older adult internet users has reached 67% [1]. In China, internet use among older adults is still in its infancy, but internet use is rapidly increasing among middle-aged and older adults [2].

As a revolutionary new technological tool, the internet provides tremendous opportunities for improving quality of life among older adults, such as through enriching their spiritual and cultural lives, maintaining and expanding their interpersonal relationships, and providing a variety of conveniences [3, 4]. Reasonable internet use benefits both the physical and mental well-being of older adults [5]. Among these benefits, reducing the depression levels of older adults through internet use is an important issue.

Both theoretical and empirical studies tend to recognize a positive effect of internet use on reducing the depression levels of older adults, although the mechanism is uncertain and controversial in some contexts. A handful of studies have shown that online engagement can impact well-being outcomes [6]. By using the internet, older people can access more health information and resources online, which has been verified to benefit their mental health [7]. Using the internet for social purposes was associated with higher levels of life satisfaction and greater goal attainment in Americans aged 80 years and older [8]. Internet use and online activities can reduce loneliness, increase social participation and strengthen social networks, thereby decreasing the level of depression in older adults [6, 9]. One study reported that the incidence of depression among older adult internet users is significantly lower than that of nonusers by 20–28% [10]. A study targeting older Korean adults shows that internet use can improve the life satisfaction of older people and thus reduce their level of depression [11]. Interventional studies on the use of the internet or computers have shown their effects on improving mental health and reducing depression in older adults [12, 13]. Additionally, based on the 2013 China Comprehensive Social Survey (CGSS) data, internet use has a positive impact on the physical and mental health of older adults [14]. Based on the 2013 China Social Situation Survey (CSS) data, internet use has been found to be beneficial in improving the life satisfaction of older adults [15]. The life satisfaction of older adults is directly related to their depression [16–18].

However, some studies have also found inconsistent results. A study targeting older Chinese adults shows that the frequency of internet use has no significant effect on mental health [19]. Huang even found a negative correlation between internet use and mental health through meta-analyses [20]. One survey of community-dwelling Canadians aged 60 years and older found that greater internet use for communication was negatively correlated with depression, while there was no significant relationship between use for entertainment and psychological outcomes [21]. Therefore, there is still not enough evidence to evaluate the relationship between internet use and depression in older adults.

One of the reasons for the lack of powerful empirical evidence on the impacts of internet use on depression levels in older adults is selection bias. Selection bias occurs when a sample is not representative of the population because of the method of selection, thus affecting the study results [22]. Obviously, there are some natural differences between older people who use the internet and those who do not. The former tend to have a higher educational level, have higher economic status, and be younger in age. In particular, the proportion of older adults who use the internet is still low in China. For example, in the sample of national households used in this study, internet users accounted for only 6.5% of the total older adult group, which exacerbated the selection bias. To overcome this problem, this study used propensity score matching (PSM) to empirically test the impact of internet use on depression levels in older adults. PSM is a statistical technique that can effectively reduce selection bias due to confounding variables between two different groups, such as the number of internet users and non-internet users among older adults. Therefore, internet use could be considered a form of treatment, and the PSM method can achieve a form of control via statistical matching in the observational data.

The propensity score matching method is based on the Neyman-Rubin counterfactual framework to automatically control for the confounding variables. Therefore, the selection of suitable confounding variables is the basis for applying PSM. In this study, confounding variables were the variables that could predict both the depression level and internet use among older adults.

The existing literature suggests that a series of social factors are associated with depression in older adults. Loneliness is recognized as one of the important factors affecting depression in older adults [23, 24]. A significant interaction has been found between loneliness and depression in older adults [24]. Some scholars have tried to explore the impact of social participation on depression in older adults. Croezen, Avendano, Burdorf, and Lenthe believe that the extent and direction of the impact depend on the type of social participation, and participation in religious activities can reduce the degree of depression in older adults [25]. Studies have also noted that although depression corresponds to decreased social participation, strong family ties with spouses and children will also help alleviate depression in older adults [26]. In addition, some researchers have focused on physical exercise. According to a meta-analysis of depression in older adults and physical exercise, physical exercise can safely and effectively alleviate depression in older adults [27]. Another meta-analysis found that structured physical exercise suitable for older adults based on ability would reduce the severity of depression [28].

In addition to the above social factors, a large number of studies have found many demographic variables and economic factors associated with depression in older adults. First, age has long been associated with older adults’ depression, as the corresponding deterioration of physical and psychological functions affects depression. Clinical studies have found that depression in older adults is related to cognitive impairment, physical inactivity, and anxiety due to their older age [29]. Second, there are gender differences in depression in older adults. For example, Djukanović, Sorjonen, and Peterson studied 9698 Swedish adults between the ages of 65 and 80 and found that depression was less common among older women [30]. In addition, educational background is also related to depression. Hoogendijk et al. conducted a 13-year follow-up study of older people over 65 years of age in the Netherlands and found that those with higher educational status showed lower levels of depression, and factors such as low income and decreased cognitive ability can reduce the inhibitory effect of education on depression, in which chronic diseases play the most important role [31]. In addition, residence has also been found to have a relationship with depression. A study of older adults in Quebec, Canada, found that older people living in rural areas had a higher rate of depression than those living in cities and metropolitan areas [32]. Next, economic factors such as income are often found to be associated with depression. Patel et al. noted that psychological stress is the mechanism by which income affects individual depression [33]. Low income increases individual anxiety and thus increases the risk of depression. Hubbard et al. studied 3625 older adults aged 65 to 79 years in England and found that income level has no significant effect on their mental health, while a higher income level may inhibit the negative impact of fragility [34]. Moreover, physical health is often connected with depression in older adults. Difficulties in physical function can exacerbate depression [35]. In addition, intelligence levels, such as cognition, are also associated. Rock et al. suggest that cognitive impairment is an important issue related to depression [36]. Finally, some researchers believe that depression is significantly associated with a lower quality of life [37]. The life satisfaction of older adults is also found to be directly related to their depression [16–18]. In other words, it can be concluded that the reason for depression among older adults is very complex.

The internet is not only an emerging high-tech product but is also the technical basis of a new lifestyle. Many scholars are very interested in why and how people use the internet [6, 38, 39]. Davis first proposed the technology acceptance model, explaining why people use the internet from the perspectives of perceived ease of use and perceived usefulness [40]. Later, the model was continuously expanded and involved new factors [41], and many different theories have been developed [42]. Some theories have been applied to Chinese older adults’ internet use, such as the technology acceptance model [43], planned behavior theory [44], and the expectation confirmation theory [45]. The unified theory of acceptance and use of technology (UTAUT) is the more influential and comprehensive theory proposed based on multiple theories [42]. In the UTAUT, factors such as sociodemographic factors, physical health factors, intellectual factors, cognitive factors, environmental factors, and social factors are all incorporated into the explanation of older adults’ internet use behaviors. A large number of empirical studies have also found that the factors affecting the internet use behavior of older adults are complex and diverse. Physical, psychological and social factors all affect the internet use of older adults [46], with physical and psychological factors being the primary factors affecting internet use in this age group. Physical and cognitive declines can block older people’s internet use behavior [47]. Both physical and mental health impact the use of technology by older people [48]. Research on internet usage behavior among older adults in China has also found various influencing factors. Zhang and Chen found that sociodemographic factors, economic conditions, physical conditions, and social support can affect older adults’ use of new forms of media [49]. Chen and Wang found that older adults’ main reasons for using the internet include spending time with others, keeping in touch with relatives, making new friends, and resocializing [50]. Additionally, Wu found that age, income, and living in urban or rural areas affect whether older people choose to use the internet [15].

According to the above literature review, we identified more than ten confounding variables between depression levels and internet use in older adults. These confounding variables resulted in selection bias in observational data, which we could not use to directly draw convincing conclusions regarding whether internet use affects depression levels in older adults. However, the propensity score matching method may provide more favorable methodological improvements to make more powerful causal inferences in observational data [22].

This study examined internet use as a treatment, with older adults using the internet as a treatment group in representative cross-sectional data. PSM methods could match older adults not using the internet in the same data as the treatment group and select a control group that is balanced with the treatment group. This approach would prevent selection bias between the treatment group and the control group. Then, computing the average treatment effect on the treated (ATT) between the treatment group and the control group could provide more powerful evidence to verify the impact of internet use on depression levels in older adults.

According to the above literature review and variable availability, this study comprehensively selected ten confounding variables as covariates of PSM, including gender, age, living in urban or rural areas, marital status, living conditions, pension status, education background, physical health, life satisfaction and intelligence.

*Hypothesis: Internet use can significantly reduce levels of depression in older adults after PSM is used to balance the selection bias between the treatment group and the control group.*

## Methods

### Sampling

The data for this study came from the China Family Panel Studies (CFPS). In order to serve the research needs, the CFPS, initiated by the Beijing University, is an almost covering the whole country, comprehensive, highest-quality, and longitudinal social survey in Chinese society. By collecting extensive information about individuals, families and communities as well as all family members, the CFPS has achieved to track the social change in Chinese society and economy [51]. Consistent with the research perspective, the questionnaires have multiple levels and across multiple domains covering on the investigations on rural and urban issues, migrant mobility, historical events in specify one family, individual experiences, and child development. It has been argued and promised that the data from CFPS can support academic research on contemporary China [51]. According to the purposes of the research, this study adopted the newest adult database of 2016. Among all the participants, there were 8103 participants aged 60 or older. After invalid cases were eliminated, such as those with missing values on internet use, we obtained an effective sample size of 7779 (effective rate = 96.0%).

### Measurement

#### Depression level

In the CFPS, depression was measured by 8 items on the Center for Epidemiological Studies-Depression scale (CES-D8). Older adults could use the CES-D8 scale to measure the severity and frequency of certain feelings and behaviors [52]. Respondents were asked how often they could not get going, felt sad, enjoyed life, felt lonely, were happy, slept restlessly, felt that everything was an effort, and felt depressed; these items were rated from 1 (none of the time) to 4 (all or almost all of the time). We consider depression to be a continuous phenomenon, with higher scores indicating a more depressed mood.

#### Internet use

In the Pew internet survey, internet users were defined as those who answered at least one of the following three questions: (1) how often do you surf the internet?; (2) do you often send or receive emails?; and (3) do you use computers and other mobile devices to surf the internet? [53]. In the CFPS database of 2016, there are two similar questions: (1) whether to use mobile devices to surf the internet and (2) whether to use the computer to surf the internet. Therefore, respondents who choose “yes” to at least one of the above two questions are regarded as using the internet in this study (internet use: 1 = yes vs. 0 = no). Among the 7779 individuals in the sample, 506 (6.5%) older adults used the internet.

#### Confounding covariates

We used ten potential confounding covariates between internet use and depression in older adults. They included individual demographic characteristics (gender, age, education background, living in urban or rural, marital status, and living condition), pensions, perceived health status, life satisfaction, and intelligence. Specifically, perceived health status and life satisfaction were rated on a five-point Likert scale. According to older adults’ performance in the interview, intelligence was scored by the interviewees who had received systematic training, with the lowest score being 1 and the highest score being 10. The ten confounding covariates are shown in Table 1.

**Table 1:** Covariate measurement and attributes

Covariates	Measurements	Meanings
Gender	1 = male vs. 0 = female	A dichotomous variable
Age	Range from 60 to 98	A continuous variable
Urban or Rural	1 = urban vs. 0 = rural	A dichotomous variable
Marital status	1 = marriage/cohabitation vs. 0 = single/ widowed	A dichotomous variable
Living condition	1 = living alone vs. 0 = living with others	A dichotomous variable
Pensions	1 = yes vs. 0 = no	A dichotomous variable
Education background	1: nursery 2: kindergarten 3: primary school 4: junior high school 5: senior high school 6: college or university	The higher the score, the higher the level of education background
Physical health	Range from 1 to 5	The higher the score, the worse the health status of the participants
Life satisfaction	Range from 1 to 5	The higher the score, the higher the life satisfaction of the participants
Intelligence level	Range from 1 to 10	The higher the score, the higher the intelligence level of the participants marked by interviewers

### Statistical analysis

In this study, PSM is the key statistical technique used to address the selective bias in observational data. “The propensity score is the conditional probability of assignment to a particular treatment given a vector of observed covariates” [54]. By matching the propensity score, we could select a subset from non-treatment units to be similar to the treatment units. Moreover, the propensity score considers multiple confounding variables. Therefore, PSM could reduce the selection bias due to confounding variables. Then, a kind of randomization that typically does not exist in observation data is created and used to estimate the effect of a treatment. It should be point out further the rationale of the PSM method is defined as the conditional probability of receiving treatment (as intervention here), which is adopted from Neyman-Rubin influential counterfactual framework [54]. The adjustments between the control groups and intervention groups automatically lead to the confounding covariates, as long as the researchers estimated the propensity scores. Therefore, the differences between the control groups and intervention groups could be identified as the effects of treatment and eliminating the effects of the confounding covariates [22].

PSM analyses are generally divided into two steps. The first analysis is to calculate the propensity score, the second analysis is to match according to the propensity score, and the last analysis is to estimate the Average Treatment effect on the Treated (ATT) [22]. Therefore, in order to empirically examine the association between internet use and reported depression in older adults, we conducted a two-step analytical strategy. We performed Psmatch2 in Stata 14.0 to conduct the statistical analysis. First, we estimated a binary logistic regression model using internet use as the dependent variable and ten demographic and socioeconomic factors as independent variables. According to the above literature review, the ten independent variables may all be confounding covariates between depression and internet use in older adults. Secondly, using the confounding covariates confirmed in the previous step, a propensity score analysis was conducted in order to control for potential bias. PSM method uses the concept and the terminology from the experiments (non-treatment units as control group vs. the treatment units as intervention group). In our study, the “treatment” refers to internet use of older adults. Specifically, the “control” group is composed of older adults who did not use the internet, while the “intervention” group is composed of older adults who use the internet. Furthermore, a paired t-test with the propensity-score-matched groups was conduct in order to compare the difference in depression between older adults who did not use the internet (as control group) and those who use the internet (as intervention group).

In addition, a series of tests are usually required to ensure the reliability of the results between the two steps of matching and estimating the ATT [22]. To ensure the robustness of the results, this study adopts three commonly used matching methods for matching, namely, kernel matching, radius matching, and nearest-neighbor matching, and performs relevant tests [55]. The traditional estimation of ATT will result in erroneous standard errors due to the high correlation between matched samples and the error between the estimated propensity value and the real value. The current solution is to obtain empirical standard errors by the bootstrap method [55]. In this study, the bootstrap method was used to estimate the ATT and empirical standard error of the treatment group.

## Results

### Descriptive statistics

Descriptive analyses were carried out for two different types of samples in this study, as well as for the comparison of differences between the two groups of samples (the independent sample t-test was used for continuous variables, and the chi-square test was used for classification variables). There are 506 older adults who use the internet and 7273 who do not use the internet. Table 2 shows statistically significant differences in depression levels between the two groups of older adults who do not use the internet (score = 15.811) and those who do use the internet (score = 13.468). However, there are also statistically significant differences between the two groups of older adults on the 8 covariates. Specifically, older adults who are younger, are in comparatively good health, have higher intelligence, have pensions, and have higher educational backgrounds reported more internet use and low levels of depression. This finding suggests that a causal relationship between internet use in older adults and their depression levels cannot simply be inferred.

**Table 2:** Descriptive characteristics of the total sample and the Internet use group before matching

Variables	Total sample (*N* = 7779)		Not Internet users group (*N* = 7273)		Internet users group (*N* = 506)		Between not internet users and internet users groups		
	Mean	SD	Mean	SD	Mean	SD	ΔMean	t	*p*
Depression level	15.660	5.333	15.811	5.368	13.468	4.227	2.343***	9.647	.000
Age	68.181	6.615	68.359	6.669	65.600	5.134	2.759***	9.069	.000
Physical health	3.587	1.185	3.601	1.196	3.381	0.988	.220***	4.032	.000
Life satisfaction	3.858	1.058	3.865	1.062	3.765	.990	.100*	2.098	.036
Intelligence level	5.148	1.378	5.093	1.384	5.947	.993	−0.854***	−13.560	.000
	Percent		Percent		Percent		ΔPercent	χ^2^	*p*
	=0	=1	=0	=1	=0	=1	=1		
Gender	49.99	50.01	50.79	49.21	38.54	61.46	−12.25***	28.41	.000
Urban or Rural	53.16	46.84	56.07	43.93	11.26	88.74	−44.81***	381.42	.000
Marital status	17.47	82.53	17.98	82.02	10.08	89.53	−7.51***	20.51	.000
Living condition	93.51	6.49	93.41	6.59	94.86	5.14	1.45	1.63	.201
Pensions	80.36	19.64	82.79	17.21	45.45	54.55	−37.34***	417.69	.000
Education background	–	–	–	–	–	–	***	1200	.000

(1) *SD* standard deviation. (2) * *p* < .05, ** *p* < .01, *** *p* < .001

### Propensity score

First, a logistic regression model was conducted with internet use as the dependent variable and 10 covariates as independent variables. The regression results show that the overall explanatory power of the model is good, and other covariates except gender and life satisfaction are statistically significant. These findings indicated that the model has a strong predictive effect on older adults’ internet use, as shown in Table 3. Second, the regression results were used to build a prediction model to calculate the propensity of all older adults in the sample who used the internet. The higher the propensity, the more likely the older adults are to use the internet.

**Table 3:** Logistic regression estimates of Internet use among older adults in China (N = 7779)

Variables	OR	Std. error	z	*p*	[95% Confidence Interval]
Constant	.523	.402	−.84	.399	[.116, 2.357]
Gender	1.175	.131	1.45	.148	[.944, 1.463]
Age	.909***	.010	−9.39	.000	[.890, .927]
Urban or Rural	5.625***	.862	11.27	.000	[4.166, 7.596]
Marital status	1.118	.219	.57	.568	[.762, 1.642]
Living condition	1.157	.309	.55	.585	[.686, 1.952]
Pensions	1.825***	.210	5.24	.000	[1.457, 2.286]
Education background	2.251***	.103	17.79	.000	[2.058, 2.461]
Physical health	.893*	.045	−2.27	.023	[.809, .985]
Life satisfaction	.908	.049	−1.77	.076	[.817, 1.010]
Intelligence level	1.302***	.063	5.46	.000	[1.184. 1.431]
Pseudo R^2^	31.8%				
AUC	.894				

(1) * *p* < .05, ** *p* < .01, *** *p* < .001; (2) *OR* odds ratio, *AUC* area under receiver operating characteristic line

### Matching and balanced test

We used the calculated propensity to match the 506 older adults who use the internet with the 7273 who do not use the internet. To ensure the robustness of the results, we adopt three common matching methods: kernel matching, radius matching, and nearest-neighbor matching. A total of 504 pairs of samples were successfully matched under kernel matching, 502 pairs under radius matching, and 506 pairs under nearest-neighbor matching. The overall matching success rate was nearly 100%, which indicated that the three methods had a good overall matching effect.

Caliendo and Kopeining suggested that the standard deviation of the two samples after PSM should be less than 5% and that the independent sample t-test of each covariate between the control group and treatment group should no longer be significant [56]. In this study, the independent sample t-test of the 10 covariates between the control group and treatment group was not significant after matching in all three matching samples, as shown in Table 4, which met the relevant recommended indicators.^1^ In addition, the change in the pseudo R^2^ of the regression equation before and after matching was also an important indicator. Before matching, the pseudo R^2^ of the binary logistic regression was 31.8%. However, it changed to 0.3, 0.2 and 0.1%, respectively, after matching. These results showed that the 10 covariates could not provide any effective predictive information about internet use after matching. Therefore, it passed the overall balance test.

**Table 4:** T-test of covariates between treatment and control group after matching

	Kernel Matching (504 pairs)		Radius Matching (502 pairs)		Nearest-neighbor Matching (506 pairs)	
Variables	t	*p*	t	*p*	t	*p*
Gender	−.48	.633	−.49	.627	−.19	.851
Age	−1.15	.251	−.95	.341	−.55	.582
Urban or Rural	1.35	.178	.86	.388	−.30	.763
Marital status	−.04	.966	−.13	.899	−.57	.570
Living condition	.09	.929	.13	.900	.39	.700
Pensions	.21	.835	.01	.991	.14	.885
Education background	.65	.515	.35	.728	.11	.915
Physical health	−.26	.795	−.19	.848	−.07	.941
Life satisfaction	.04	.971	−.03	.975	−.52	.601
Intelligence level	.78	.433	.63	.530	.14	.886

### Sensitivity analysis

Rosenbaum and Rubin suggested that a sensitivity analysis must be conducted [54]. Rosenbaum proposed that the objective of a sensitivity analysis is to explore to what extent the hidden selection bias can change the results of the treatment effectiveness obtained [58]. The higher the sensitivity score is, the lower the sensitivity. Generally, if the score is greater than or equal to 2, the sensitivity of the study is lower, and the results are reliable. Therefore, a sensitivity analysis was also conducted in this study after matching. A sensitivity value of 1.9 was calculated using the Rbounds module in Stata. Although it did not reach the standard of greater than or equal to 2, we think 1.9 was also within the acceptable range.

### Final results

After matching according to the propensity score, the average treatment effect on the treated (ATT) can be estimated based on the matching results. In this study, the bootstrap method was used to estimate the ATT and empirical standard error on three matching results. The estimated results show that older adults who used the internet (treatment group) showed significantly lower depression levels than did older adults who did not use the internet (control group) after PSM was used to analyze the confounding covariates. The depression levels were significantly reduced by 0.789, 0.734, and 0.562, respectively, in the three different matching methods (see Table 5).

**Table 5:** Average treatment effect on the treated in three matching methods

Matching Method	Depression level		ATT	Bootstrap SE	z	*p*
	Treated group	Control group				
Kernel Matching	11.811	12.570	−.759***	.179	−4.24	.000
Radius Matching	11.801	12.534	−.734***	.195	−3.76	.000
Nearest-neighbor Matching	11.796	12.359	−.562*	.240	−2.34	.019

(1) *SE* standard error, *ATT* average treatment effect on the treated; (2) * *p* < .05, ** *p* < .01, *** *p* < .001

## Discussion

This article aimed to gain a better understanding and empirical evidence of whether older adults’ internet use affects their depression level by adopting control and treatment groups using the PSM approach. From a theoretical perspective, first, based on the 2016 China Family Panel Study (CFPS) data, this study explores the causal effects of older adults’ internet use on their depression levels. Specifically, the group of older adults who use the internet had a lower level of depression. In contrast, the group of old adults who do not use the internet had a higher level of depression. This result appeared to confirm that internet use could significantly affect depression levels in older adults after adjustments were made for ten key confounding covariates with PSM. Thus, this study provides more powerful evidence among many related studies on this issue. The meta-analyses of Huang found that the results of correlations between internet use and psychological well-being were heterogeneous and not statistically significant for older adults [20] because it is difficult to establish the causal effect of internet use on the mental health of older adults. This study obtained an ideal control group through exact matching. It made the samples of the treatment group and the control group meet the basic requirements of causal inference. The positive impact of internet use on the mental health of older adults in this study is approximately 21.3–28.4% of the results of Wang, who used an ordered logistic regression model based on the 2013 CGSS data [14]. This finding suggests the use of the PSM approach to eliminate the greater impact of confounding factors. In this way, this study provides more solid evidence of older adults’ internet use on reducing their depression levels. To some extent, this study resolves the inconsistent conclusions of previous studies on this issue.

From a methodological perspective, this study adopted a new approach to further understand and verify the impact of internet use on depression levels. Traditionally, causal inference can only be achieved through experimental research, overcoming selection bias with random grouping, or at least a longitudinal study. In contrast, observational research based on survey data is unable to control random grouping and thus faces severe challenges of selection bias; as a result, it is difficult to obtain convincing evidence of causal inference. As an intuitive, convenient solution to selection bias, the PSM method adopted in this study is gaining increasing attention in various fields of social science. With PSM, a quasi-experimental study was constructed in which both the treatment group and the control group sample reached 504 and thus achieved a relatively reliable and convincing causal inference—older adults in China can significantly reduce depression levels through internet use.

A perfect group of confounding covariates is the basis of exact matching. This study identified ten confounding covariates from a literature review, including demographic factors (gender, age, education background, living in urban or rural, marital status, and living condition) and socioeconomic factors (pensions, perceived health status, life satisfaction, and intelligence). The results of logistic regression showed that the overall explanatory power is very good and that all confounding covariates significantly predicted internet use among older adults except gender, marital status, living condition and life satisfaction. The confounding covariates also showed consistent impacts on older adults’ internet use with theoretical predictions. The results of no significant difference between all three matching control groups and treatment groups further indicated that the control groups and the treatment groups were well balanced. Overall, this study eliminated selection bias. Therefore, this study made a causal inference between older adults’ internet use and their depression levels.

The proportion of older adults in China using the internet is still very low. Therefore, this study also has important policy implications for encouraging older adults to use the internet as an intervention for their depression. This study provides relatively direct, powerful and practical evidence. In fact, there have been many social service programs offered by local governments, communities, universities for the aged, and NGOs to help older adults use the internet. However, the goals of these programs are diverse, including popularizing internet use, improving the quality of life of the elderly, and broadening social life for the elderly. Based on this study, the alleviation of depression could also be added to such social services. In addition, internet use may serve as an adjuvant therapy for older adults suffering from depression.

### Limitations

There are some limitations to this study. First, the ten covariates used in this study may not cover all the important confounding factors between internet use and depression in older adults. On the one hand, this study used second-hand survey data, which is inevitably limited in terms of variable selection. On the other hand, the factors affecting older adults’ internet use and depression are very complicated and diverse, and it is too difficult and unnecessary to cover all factors. The ten covariates selected in this study covered the main aspects affecting older adults’ internet use and depression and seemed adequate. Second, apart from the PSM method, other statistical techniques, such as regression adjustments and instrumental variables, are also used to address selective bias. Cross-validation with a variety of methods will surely increase the reliability of the conclusion. Third, internet use behavior among older adults is complicated, while this study only distinguishes between using and not using the internet; furthermore, it impossible to explore what kind of internet use behavior is more effective for depression alleviation. These limitations are worthy of more relevant future studies.

## Conclusions

Our results indicate that older adults in China using the internet have significantly lower levels of depression than those who do not use the internet after confounding covariates were excluded through the application of PSM. This is more solid evidence because the PSM approach is for cases of causal inference. In light of the conclusions of this study, society should provide more support and convenience for older adults to use the internet in the future. For example, social workers’ provision of training on internet use for older adults and the provision of special places for older adults to use the internet in the community will help promote internet use among older adults.

## Data Availability

The data that support the findings of this study are available from [Institute of Social Science Survey, Peking University] but restrictions apply to the availability of these data, which were used under license for the current study, and so are not publicly available. Data are however available from the authors upon reasonable request and with permission of [Institute of Social Science Survey, Peking University].

## Abbreviations

ATT: Average treatment effect on the treated
CFPS: China Family Panel Study
CGSS: China Comprehensive Social Survey
CSS: China Social Situation Survey
NGOs: no*n-go*vernmental organizations
PSM: Propensity score matching
TAM: Technology acceptance model
UTAUT: Unified theory of acceptance and use of technology

## Footnotes

1 In theory, only the continuous variables could be tested for balance here. However, the independent sample t-test also could provide enough information for the balance test for dichotomous and categorical variables [57].
